# Acute Q fever in patients with an influenza-like illness in regional New South Wales, Australia

**DOI:** 10.1371/journal.pntd.0012385

**Published:** 2024-08-05

**Authors:** Chaturaka Rodrigo, Gregory Walker, Andrea T. K. Sevendal, Chelsea Nguyen, Sacha Stelzer-Braid, William Rawlinson, Stephen Graves, Heather F. Gidding, John Stenos, Andrew R. Lloyd

**Affiliations:** 1 School of Biomedical Sciences, Faculty of Medicine and Health, University of New South Wales, Sydney, New South Wales, Australia; 2 NSW Health Pathology, Prince of Wales Hospital, Randwick, New South Wales, Australia; 3 Australian Rickettsial Reference Laboratory, Geelong, Victoria, Australia; 4 School of Population Health, Faculty of Medicine and Health, University of New South Wales, Sydney, New South Wales, Australia; 5 Kirby Institute, Faculty of Medicine and Health, University of New South Wales, Sydney, New South Wales, Australia; Postgraduate Institute of Medical Education and Research, INDIA

## Abstract

**Introduction:**

Query (Q) fever is a zoonosis caused by the bacterium *Coxiella burnetii* typically presenting as an influenza-like illness (ILI) with or without hepatitis. The infection may be missed by clinicians in settings of low endemicity, as the presentation is clinically not specific, and there are many more common differential diagnoses for ILI including SARS-CoV-2 infection.

**Methods:**

Residual serum samples were retrospectively tested for Phase 1 and 2 Q fever-specific IgM, IgG, IgA antibodies by indirect immunofluorescence and *C*. *burnetii* DNA by polymerase chain reaction. They had not been previously tested for Q fever, originating from undiagnosed patients with probable ILI, aged 10–70 years and living in regional New South Wales, Australia. The results were compared with contemperaneous data on acute Q fever diagnostic tests which had been performed based on clinicians requests from a geographically similar population.

**Results:**

Only one (0.2%) instance of missed acute Q fever was identified after testing samples from 542 eligible patients who had probable ILI between 2016–2023. Laboratory data showed that during the same period, 731 samples were tested for acute Q fever for clinician-initiated requests and of those 70 (9.6%) were positive. Probability of being diagnosed with Q fever after a clinician initiated request was similar regardless of the patients sex, age and the calendar year of sampling.

**Conclusion:**

In this sample, Q fever was most likely to be diagnosed via clinician requested testing rather than by testing of undiagnosed patients with an influenza like illness.

## Author summary

Q fever is a highly contagious infection presenting as an influenza-like illness or less commonly as acute hepatitis. Literature shows that there may be diagnostic delays in recognizing Q fever by primary care physicians if they are unfamiliar with the infection. We tested residual serum samples stored at NSW Health Pathology between 2016–2023 for Q fever, if that sample originated from a rural postcode in NSW and had been tested previously for an organism causing influenza like illnesses, but not Q fever. After testing 542 eligible samples only one case of missed acute Q fever was found. In the same period, from a similar population the same diagnostic laboratory received 731 requests for Q fever testing from which 70 were positive. Therefore, In this study the number of missed acute Q fever cases were clinically insignificant.

## Introduction

Query (Q) fever is a zoonosis caused by the highly infectious intracellular bacterium *Coxiella burnetii*, and domesticated cattle, goats and sheep are the typical animal reservoir of the pathogen. Most Q fever infections (60%) are minimally symptomatic or asymptomatic [1]. In symptomatic disease, acute infection predominantly manifests as an influenza-like illness (ILI) with or without hepatitis, and less commonly as pneumonia, cardiac (pericarditis, myocarditis and endocarditis) and neurological disease.[2] Chronic localised infection with Q fever occurs in a minority of patients manifesting as endocarditis, infection of prosthetic vascular grafts, or vertebral osteomyelitis. The main transmission mode in humans is via inhalation of aerosols, with direct innoculation and ingestion occasionally reported. Those in contact with domesticated livestock and with wild animals such as farmers, abattoir workers, pig shooters, butchers, wildlife and forestry workers, and veterinarians are at particularly high risk of infection. [2] The largest outbreak of Q fever reported to date is from the Netherlands with approximately 4000 cases (from an estimated 45,000 total infections) reported between 2007 and 2010, arising from aerosol transmission from infected herds of domesticated goats. [3]

In Australia, a Q fever vaccine is recommended by the Australian Technical Advisory Group (ATAGI) for people above 15 years of age. [4,5] However that the product information for Q fever vaccine states that the vaccine is approved for immunisation of susceptible adults at identifiable risk of infections with Q fever. The vaccine is highly effective, [6] but has a significant rate of both local and systemic delayed type hypersensitivity reactions particularly occurring in people with pre-existing exposure to *C. burnetii* and probable immunity. Hence, pre-administration serological and skin testing is necessary to exclude individuals who may be at a high risk of severe adverse reactions. A nationally funded Q fever vaccination programme was initiated in Australia in 2002 for certain high-risk groups with a 100% coverage within abattoir workers (and a less impressive 43% coverage among farmers), but it is no longer funded. During the campaign, notifications and hospitalisations due to Q fever declined by 50%. [7] Following cessation of the immunisation program, estimates in 2014 indicate that the vaccination rates now vary between 30–75% in high risk groups [8]. It is likely that Q fever infection rates will rise as the prevalence of vaccine uptake falls in the high turnover occupational risk groups in places where there is no formalised vaccination program. Thus, awareness of Q fever infection and its consideration as a differential diagnosis in primary care is critical both for reliable epidemiological estimates and also for individual patient care.

Inclusion of Q fever in the differential diagnosis of an ILI may not always be considered unless health-care workers are alert to its presence. Out of 2238 patients diagnosed with acute Q fever in Queensland, Australia from 2003 to 2017, 53% were hospitalised, with a 5-day median hospital stay (and a 10-day median of absence from work) [9]. As Q fever infection is associated with a high subclinical to clinical ratio, [1] this hospitalisation rate suggests a strong bias towards consideration of acute Q fever only in those with severe illness. In the Netherlands, GPs who had seen eight or more cases of Q fever were significantly less likely (rate ratio 0.67) to have diagnostic delay than those who had seen only one case previously (95% CI: 0.59–0.76), suggesting familiarisation with the diagnosis and testing approach is often acquired by experience [10]. These figures are consistent with diagnostic delays observed elsewhere in a non-epidemic, low-incidence context. For example, in a single centre in South Korea, the average delay in diagnosis (for over a 4-year period (2015–2018) was 21 days. [11] In New South Wales, Australia Q fever is currently not routinely included in the multiplexed serology or PCR diagnostic panels used to investigate ILIs and after the pandemic SARS-CoV-2 infection is more likely to be considered as a differential diagnosis than Q fever.

We hypothesized that a significant proportion of patients from regional New South Wales, Australia undergoing serological testing for a pathogen likely to cause ILI or atypical pneumonia may have unsuspected acute Q fever. Therefore, this study aimed to estimate the prevalence of missed Q fever by retrospectively testing serum from undiagnosed patients with a plausible influenza like illness, living in regional NSW. It also aimed record the number of diagnosed Q fever cases from a similar demographic during the corresponding period for comparison.

## Methods

### Ethics statement

This study was approved by the South Eastern Sydney Local Health District Human Research Ethics Committee of New South Wales Health (2021/ETH10995).

### Clinical samples

Clinical samples for this study were received from NSW Health Pathology’s Randwick Campus (South Eastern Sydney Local Health District) and consisted of residual serum from patients who were likely to have been referred for diagnostic testing due to an ILI or atypical pneumonia and where all diagnostic tests were negative. There was no direct recruitment of patients and because microbiology request forms did not always indicate the clinical presentation, eligible samples were selected based on the profile of tests requested. Thus de-identified residual serum samples received from individuals residing in regional area postcodes of NSW (2250–2308, 2311–2490, 2500–2551, 2574–279, 2753–2754, 2756–2758, 2773–2898)[12], and aged 10–70 years were selected (one sample per patient), if a diagnostic test was requested and tested negative for any of the following pathogens: Rickettsial species, *Bordetella pertussis*, SARS-CoV-2, Chlamydia group, *Legionella longbeachae* or *L pneumophila*, *Mycoplasma pneumoniae*, Influenza A and B, and Parainfluenza 1, 2 or 3 virus infections. When a diagnostic test for Q fever was requested for an eligible patient, the sample was not retrieved, but the result of the test was extracted for analysis. Samples that had not been tested for any of the pathogens listed above, those that tested positive for one or several of above pathogens, samples from patients residing outside of regional NSW or outside of the age group of interest as well as multiple samples from same patient were excluded. The samples were de-identified by NSW Health Pathology and only the following data were linked to each tested sample: age at the time of sampling, postcode of residence, specimen collection year and sex of patient.

### Sample size

The estimates for previous Q fever infections in the broader community in Australia vary from 2–5% [13,14] but can be as high as 10% in high-risk subgroups in rural areas, based on previous seroprevalence studies in Australia by Gidding et al [13]. Therefore, we considered an estimate of 10% for this study in regional populations where the epidemiological risk is higher. If the maximum prevalence of missed Q fever is expected to be 20% and the study assumes a desired 95% CI of +/-5%—the minimum sample size needed was 246 specimens (excluding specific requests for Q fever). The expected point estimate of 10% would provide even tighter confidence intervals.

### Diagnosis of Q fever

All selected samples were tested for Q fever Phase 1 and Phase 2 IgA, IgG and IgM antibodies by indirect immunofluorescence assays and for *Coxiella burnetii* DNA by real-time Polymerase chain reaction (PCR, gene targets—com1 and htpAB). Seroconversion in acute Q fever usually occurs between day 7–15 post symptom onset and almost always by day 21. PCR is often positive in symptomatic Q fever patients before seroconversion [15] and with this suite of tests, it is unlikely any acute Q fever cases were missed. The samples were shipped in dry ice to the Australian Rickettsial Reference Laboratory, Geelong, Victoria (VIC), Australia for testing. A sample was considered as positive for acute Q fever when *C*. *burnetii* DNA was detected by PCR and/or phase 2 IgM was detected and/or seroconversion was detected on serial sampling (4-fold rise in phase 2 IgG) and / or when having a single IgG titre > 1:128 according to the Public Health Laboratory Network case definitions for acute Q fever diagnosis [16].

Concurrently, the data received on Q fever tests already performed by NSW Health Pathology showed that Phase 1 and Phase 2 IgG and IgM assays (and occasionally IgA) had been done routinely when requested, but not PCR. In addition, as serial samples from the same patient were not collected for this study, diagnosing based on seroconversion of paired samples was only relevant to patients already tested by NSW Health Pathology.

### Data analysis

Descriptive data are presented with measures of central tendency (mean, median, mode) and dispersion (standard deviation. Inter-quartile range), as appropriate. Both the missed and diagnosed Q fever numbers were intended to be compared between subgroups of sex, geographical location and year of sampling (as Q fever prevalence may increase in years with low rainfall) but this could not be done due to the low number of missed infections (see results). Instead, a subgroup analysis was performed for diagnosed Q fever cases to see if any of the above subgroups had a higher likelihood of being diagnosed with Q fever. For geographical location, adjacent postcodes were collapsed as Northern (New England and Northwest, North coast), Southern (Southeast and Tablelands including ACT, Illawarra-Shoalhaven), Central Coast plus Hunter, and Western (rest of NSW excluding greater Sydney) regions of NSW for analysis. Eligible ACT postcodes were included in the analysis as ACT is entirely within NSW. The chi-square test and independent *t* test were used for between group comparisons of categorical and continuous data, respectively. Statistical significance was set at p<0.05.

## Results

A total of 542 residual serum samples that met the inclusion criteria were tested (Table 1). These non-identifiable samples were acquired retrospectively in five rounds between 2021–2023, at 6–12-month intervals. The mean age of patients was 47.6 years (SD ± 17.4 years, range: 10–70) and 291 (53.7%) were males. The samples were originally collected between calendar years 2016–2023 but a disproportionately higher number of samples were from calendar years 2022 (263, 48.5%) and 2023 (209, 38.6%) as NSW Pathology had discarded most of their historical samples from cold storage during the pandemic. Most samples were from the Southern regional postcodes of NSW (485, 89.5%), followed by Northern (25, 4.6%), Western (17, 3.1%) and Central coast and Hunter (15, 2.8%) regions (S1 Table). From all samples tested four recent Q fever infections were identified (0.74%, 95% CI: 0.29–1.88%) but only one could be ruled in as a missed acute Q fever infection responsible for the current episode of illness, with certainty (1/542, 0.18%, 95% CI: 0.03–1.03%). Another 6 (1.1%, 95% CI: 0.51–2. 4%) had evidence of past infections.

**Table 1:** Comparison of two groups that were tested for missed Q fever (Group 1) and those referred for Q fever testing based on clinical suspicion (Group 2).

Variable	Group 1 (N = 542)		Group 2 (N = 731)		P value*
	Number (%)	Mean ± SD	Number (%)	Mean ± SD	
Age (years)		47.59 ± 17.43		45.93 ± 16.69	0.085
Age categories (years)					
*10–29*	110 (20.3)		147 (20.1)		0.920
*30–49*	139 (25.6)		231 (31.6)		0.021
*50–70*	293 (54.1)		353 (48.3)		0.042
Sex					
*Males*	291 (53.7)		479 (65.5)		<0.001
*Females*	251 (46.3)		252 (34.5)		<0.001
Year of sample collection**					
*2016*	7 (1.3)		104 (14.2)		<0.001
*2017*	4 (0.7)		82 (11.2)		<0.001
*2018*	0 (0)		75 (10.3)		<0.001
*2019*	1 (0.2)		69 (9.4)		<0.001
*2020*	14 (2.6)		49 (6.7)		<0.001
*2021*	44 (8.1)		146 (20.0)		<0.001
*2022*	263 (48.5)		122 (16.7)		<0.001
*2023*	209 (38.6)		84 (11.5)		<0.001
Geography					
*Central Coast and Hunter*	15 (2.8)		10 (1.4)		0.075
*Northern regional*	25 (4.6)		71 (9.7)		<0.001
*Southern regional*	485 (89.5)		611 (83.6)		0.002
*Western regional*	17 (3.1)		39 (5.3)		0.059
Samples diagnosed with acute Q fever	1 (0.2)		70 (9.6)		<0.001

*P values calculated with chi square test (Fishers exact test was used when appropriate) or independent *t* test

**The statistical differences are an artifact because NSW Health Pathology had discarded many historical samples collected from 2016 to 2020 from storage due to pandemic related disruptions, and hence they were unavailable to be tested within group 1.

During the corresponding period (2016–2023), samples from 731 patients living in regional NSW had been referred to NSW Health Pathology for Q fever testing based on clinical suspicion (Table 1). In this cohort, the mean age was 45.9 years (SD: ± 16.7 years, range: 10–70) with 479 (65.5%) males. The highest number of samples were tested in 2021 (146, 20%) and the lowest in 2020 (49, 6.7%). Most samples had been referred from Southern regional postcodes of NSW (611, 83.6%), followed by Northern (71, 9.7%), Western (39, 5.3%) and the Central Coast and Hunter region (10, 1.4%, S2 Table). The differences in demographics between the two groups (whom we tested for missed Q fever, and those tested based on clinicians’ request) are summarized in Table 1. Both groups had a similar mean age and geographical distribution, but there was a higher proportion of males in the group that were clinically suspected to have Q fever. Similarly, when compared in 20-year age brackets, group tested for Q fever by us had proportionately more individuals aged 50–70 years. The differences in calendar year of sampling between the two groups are an artifact due to the reasons mentioned above. Overall, 70 (9.6%, 95% CI: 7.65–11.93%) patients tested positive for acute Q fever in the group that were referred for testing based on clinical suspicion, a number statistically significantly higher than the missed infections during the same period (p<0.0001, Chi square test). A subgroup analysis of Q fever positive patients (Table 2) showed similar probability of testing positive for acute Q fever regardless of age, sex, calendar year of sampling, and geographical location (except Southern regional postcodes having a higher probability than rest of regional NSW).

**Table 2:** Subgroup differences among patients testing negative or positive for acute Q fever following referral by a clinician for testing (N = 731).

Variable	Negative for Q fever (n = 661)	Positive for Q fever (n = 70)	P value*
	Number (%) or mean ± SD	Number (%) or mean ± SD	
Age (in years)	45.84 ± 16.89	46.87 ± 14.97	0.59
Age categories (years)			
*10–29*	137 (20.7)	10 (14.3)	0.202
*30–49*	209 (31.6)	22 (31.4)	0.920
*50–70*	315 (47.7)	38 (54.3)	0.292
Sex			
*Male*	427 (64.6)	52 (74.3)	
*Female*	234 (35.4)	18 (25.7)	0.105
Year of sampling**			
*2016*	97 (14.7)	7 (10)	0.288
*2017*	77 (11.6)	5 (7.1)	0.322
*2018*	68 (10.3)	7 (10)	0.92
*2019*	60 (9.1)	9 (12.9)	0.303
*2020*	45 (6.8)	4 (5.7)	0.81
*2021*	127 (19.2)	19 (27.1)	0.115
*2022*	113 (17.1)	9 (12.9)	0.365
*2023*	74 (11.2)	10 (14.3)	0.442
Geographical location**			
*Northern regional*	68 (10.3)	3 (4.3)	0.107
*Southern regional*	546 (82.6)	65 (92.9)	**0.04** ^#^
*Western regional*	37 (5.6)	2 (2.9)	0.417
*Central coast and Hunter*	10 (1.5)	0 (0)	Not applicable

*P values calculated with chi square test (Fishers exact test was used when appropriate) or independent *t* test

**2 x 2 table comparisons were done using positive and negative cases within group vs. positive and negative cases outside of that group (e.g., 2016 samples vs. non-2016 samples).

^#^Statistically significant at p < 0.05.

Finally, almost half the samples (352/731, 48.2%) tested for Q fever by NSW Health Pathology were from the period (2021–2023) when we were also collecting samples for missed Q fever testing. However, NSW Health pathology did not diagnose significantly more acute Q fever cases in 2021–2023 compared to 2016–2020 (p = 0.279, chi square test) or when compared to the 3 years (2018–2020) immediately preceding the start of the study (p = 0.888, chi square test).

## Discussion

This serological analysis of 542 residual samples from patients predominantly residing in southern regional NSW with likely ILI identified only one case of missed Q fever. On the contrary, when Q fever testing was requested by the treating physician in a similar population, almost one in ten people had a positive test result. Therefore, clinical suspicion and diagnosis of acute Q fever in symptomatic patients within this sample was satisfactory and missed acute Q fever was not a clinically significant problem.

The “typical” symptoms in acute Q fever shows a geographic variation. In one of the largest retrospective analyses of Q fever clinical manifestations conducted in a tertiary referral centre for Q fever in France from 1985–98 (n = 1070), evidence of biochemical hepatitis (with fever) was seen in 40% of patients, while pneumonia was seen in 17%. An additional 17% of patients had isolated fever. Younger and older patients were more likely to present with hepatitis and pneumonia respectively. [17] Smaller case series have reported the main clinical findings to be fever alone (seen in >80% of Q fever patients in South Korea)[18], or fever with hepatitis (Israel and Portugal) [19,20], or pneumonia (Serbia) [21]. The “hepatitis” in Q fever is predominantly an elevation of liver enzymes in febrile patients with some associated nausea and anorexia, while overt jaundice is rare [22]. Previous studies from Australia show ILI to be the most common manifestation, with pneumonia seen in only 0–10% of all diagnosed patients, and hence we focussed on samples tested for pathogens causing an ILI, but not for *C*. *burnetii*. [23–26] The ILI associated with Q fever is characterised by fever of abrupt onset, chills and rigors, myalgias, headaches, and fatigue (with a normal chest X ray). [1,17] In lower incidence settings, there are many other, more common, differential diagnoses for an ILI that primary care practitioners may consider other than Q fever with SARS-CoV-2 infection recently being added to the list. [27]

The studies mentioned above analyse the symptomatology of diagnosed Q fever patients. The reverse design, that is screening patients for “missed” Q fever when having an influenza like illness or a combination of epidemiological risk factors and clinical features, is rare. One study in Iran, screened 116 people for Q fever because they had a non-specific febrile illness and a history of recent contact with livestock, and found the prevalence of acute Q fever to be 13.8% (95% CI: 8–21) [28]. Screening of 1067 patients with acute fever or pyrexia of unknown origin in North-eastern Kenya revealed acute Q fever in 16.2% of patients (95% CI: 14.1–18.7) and this diagnosis was not suspected by any of the treating physicians [29]. In Japan, screening of 400 patients with community acquired respiratory tract infections, identified 10 (2.5%) patients with Q fever. [30] There were no previous studies of similar design in Australia.

Requests for testing and the rate of diagnosis are better in settings where health care practitioners are familiar with Q fever. Australian healthcare workers may be more familiar with Q fever because the illness itself was first described in Australia after an outbreak of abattoir fever in Brisbane [31]. Later, Australian, and American microbiologists independently linked this new clinical syndrome to *C*. *burnetii*, a rickettsia like organism. Australia is also the only country that have licensed a Q fever vaccine for humans [32]. Thus, general practitioners and hospitals in regional NSW may readily consider Q fever as a differential diagnosis in appropriate clinical contexts due to their familiarity with the infection, thus keeping the missed cases to a minimum.

This study had several limitations. First, detailed clinical descriptions on the microbiology request forms were not available, so eligibility was based on the pathology tests requested. Second, the NSW Health Pathology microbiology lab of Southeastern Sydney local health district receives a higher proportion of samples from Southern regional postcodes and in particular from the Illawara-Shoalhaven region. Therefore, all regional NSW postcodes were not equally represented in this sample. Specially the Northern and Western NSW rural postcodes that have a higher reported case incidence were underrepresented in this study [33]. The inclusion of NSW Health Pathology labs from other local health districts was difficult when balancing additional governance requirements with the timeline of the project. Furthermore, some of the samples might have originated from temporary residents within Sydney metropolitan area (e.g. visitors, tourists) who have indicated their usual postcode of residence as a regional postcode. We could not exclude such samples from the information available to us. Third, NSW Health Pathology was aware that this study had commenced from 2021 which could have resulted in more Q fever tests than usual. However, the probability of detecting acute Q fever by NSW Health Pathology during the 3 years of the study (2021–2023) was not any different to that of the 3 years preceding the study (2018–2020). Fourthly, the Q fever diagnostic tests done for this study were slightly different to that done routinely by the NSW Health pathology. For example, *C*. *burnetii* PCR was not done for any of the samples tested in NSW Health pathology and paired serum testing done by NSW Health Pathology could not be done for any of the samples of this study. This effects direct head-to-head comparison of positive results. Finally, not all regional areas of NSW have a similar incidence of the disease and the samples tested for missed Q fever in this study did not originate from known hotspots for Q fever [33,34]. Similarly for the diagnosed group with Q fever, we are unaware how many people with Q fever symptoms were not tested. Given these biases, it is not possible to generalise our findings to whole of regional NSW.

## Conclusion

The prevalence of missed Q fever identified in this study in patients with a probable ILI, aged 10–70 and living mostly in southern regional NSW was less than 1:500. In contrast, when Q fever tests were requested based on clinical suspicion from a similar group of patients, nearly 1 in 10 patients returned a positive result. Therefore, in this study Q fever was most likely to be diagnosed via a clinician-initiated request than by non-specific testing of samples from undiagnosed patients with an influenza like illness. Hence, the hypothesis of a significant proportion of missed Q fever infections was rejected for this sample.

## Supporting information

S1 Table Regional NSW postcodes from which samples were tested for Q fever in this study. (DOCX)

S2 Table Regional NSW postcodes from which samples were tested for Q fever by NSW Health Pathology lab during the same period. (DOCX)
