# Supplementary material for: Acute Q fever in patients with an influenza-like illness in regional New South Wales, Australia
Source: PLoS Negl Trop Dis. 2024 Aug 5;18(8):e0012385. doi: 10.1371/journal.pntd.0012385 (PMC11326637; doi:10.1371/journal.pntd.0012385)
Supplement: S1 Table — (DOCX) [file pntd.0012385.s001.docx]

S1 Table. NSW postcodes from which samples were tested for Q fever in this study

|  | | | | | |
| --- | --- | --- | --- | --- | --- |
| Postcode | | Samples received | Percent | Valid Percent | Cumulative Percent |
| Valid | 2250 | 2 | .4 | .4 | .4 |
|  | 2251 | 1 | .2 | .2 | .6 |
|  | 2259 | 2 | .4 | .4 | .9 |
|  | 2260 | 1 | .2 | .2 | 1.1 |
|  | 2263 | 1 | .2 | .2 | 1.3 |
|  | 2264 | 1 | .2 | .2 | 1.5 |
|  | 2283 | 1 | .2 | .2 | 1.7 |
|  | 2325 | 1 | .2 | .2 | 1.8 |
|  | 2327 | 1 | .2 | .2 | 2.0 |
|  | 2330 | 1 | .2 | .2 | 2.2 |
|  | 2337 | 1 | .2 | .2 | 2.4 |
|  | 2340 | 1 | .2 | .2 | 2.6 |
|  | 2343 | 1 | .2 | .2 | 2.8 |
|  | 2345 | 1 | .2 | .2 | 3.0 |
|  | 2370 | 2 | .4 | .4 | 3.3 |
|  | 2371 | 2 | .4 | .4 | 3.7 |
|  | 2372 | 1 | .2 | .2 | 3.9 |
|  | 2390 | 1 | .2 | .2 | 4.1 |
|  | 2430 | 2 | .4 | .4 | 4.4 |
|  | 2440 | 1 | .2 | .2 | 4.6 |
|  | 2443 | 1 | .2 | .2 | 4.8 |
|  | 2444 | 1 | .2 | .2 | 5.0 |
|  | 2448 | 1 | .2 | .2 | 5.2 |
|  | 2450 | 1 | .2 | .2 | 5.4 |
|  | 2452 | 1 | .2 | .2 | 5.5 |
|  | 2453 | 1 | .2 | .2 | 5.7 |
|  | 2455 | 1 | .2 | .2 | 5.9 |
|  | 2460 | 3 | .6 | .6 | 6.5 |
|  | 2470 | 1 | .2 | .2 | 6.6 |
|  | 2474 | 1 | .2 | .2 | 6.8 |
|  | 2484 | 1 | .2 | .2 | 7.0 |
|  | 2485 | 1 | .2 | .2 | 7.2 |
|  | 2488 | 1 | .2 | .2 | 7.4 |
|  | 2500 | 31 | 5.7 | 5.7 | 13.1 |
|  | 2502 | 10 | 1.8 | 1.8 | 14.9 |
|  | 2505 | 5 | .9 | .9 | 15.9 |
|  | 2506 | 7 | 1.3 | 1.3 | 17.2 |
|  | 2508 | 11 | 2.0 | 2.0 | 19.2 |
|  | 2515 | 6 | 1.1 | 1.1 | 20.3 |
|  | 2516 | 7 | 1.3 | 1.3 | 21.6 |
|  | 2517 | 5 | .9 | .9 | 22.5 |
|  | 2518 | 17 | 3.1 | 3.1 | 25.6 |
|  | 2519 | 11 | 2.0 | 2.0 | 27.7 |
|  | 2525 | 10 | 1.8 | 1.8 | 29.5 |
|  | 2526 | 25 | 4.6 | 4.6 | 34.1 |
|  | 2527 | 32 | 5.9 | 5.9 | 40.0 |
|  | 2528 | 59 | 10.9 | 10.9 | 50.9 |
|  | 2529 | 44 | 8.1 | 8.1 | 59.0 |
|  | 2530 | 44 | 8.1 | 8.1 | 67.2 |
|  | 2533 | 18 | 3.3 | 3.3 | 70.5 |
|  | 2534 | 5 | .9 | .9 | 71.4 |
|  | 2535 | 5 | .9 | .9 | 72.3 |
|  | 2536 | 2 | .4 | .4 | 72.7 |
|  | 2537 | 3 | .6 | .6 | 73.2 |
|  | 2538 | 1 | .2 | .2 | 73.4 |
|  | 2539 | 18 | 3.3 | 3.3 | 76.8 |
|  | 2540 | 50 | 9.2 | 9.2 | 86.0 |
|  | 2541 | 33 | 6.1 | 6.1 | 92.1 |
|  | 2548 | 1 | .2 | .2 | 92.3 |
|  | 2550 | 4 | .7 | .7 | 93.0 |
|  | 2575 | 1 | .2 | .2 | 93.2 |
|  | 2576 | 1 | .2 | .2 | 93.4 |
|  | 2577 | 2 | .4 | .4 | 93.7 |
|  | 2578 | 1 | .2 | .2 | 93.9 |
|  | 2580 | 6 | 1.1 | 1.1 | 95.0 |
|  | 2583 | 2 | .4 | .4 | 95.4 |
|  | 2600 | 1 | .2 | .2 | 95.6 |
|  | 2602 | 2 | .4 | .4 | 95.9 |
|  | 2607 | 2 | .4 | .4 | 96.3 |
|  | 2614 | 1 | .2 | .2 | 96.5 |
|  | 2617 | 1 | .2 | .2 | 96.7 |
|  | 2620 | 1 | .2 | .2 | 96.9 |
|  | 2650 | 1 | .2 | .2 | 97.0 |
|  | 2653 | 2 | .4 | .4 | 97.4 |
|  | 2671 | 1 | .2 | .2 | 97.6 |
|  | 2700 | 1 | .2 | .2 | 97.8 |
|  | 2711 | 2 | .4 | .4 | 98.2 |
|  | 2712 | 1 | .2 | .2 | 98.3 |
|  | 2756 | 1 | .2 | .2 | 98.5 |
|  | 2794 | 1 | .2 | .2 | 98.7 |
|  | 2800 | 1 | .2 | .2 | 98.9 |
|  | 2821 | 2 | .4 | .4 | 99.3 |
|  | 2829 | 1 | .2 | .2 | 99.4 |
|  | 2830 | 1 | .2 | .2 | 99.6 |
|  | 2844 | 1 | .2 | .2 | 99.8 |
|  | 2852 | 1 | .2 | .2 | 100.0 |
|  | Total | 542 | 100.0 | 100.0 |  |
