# Supplementary material for: Acute Q fever in patients with an influenza-like illness in regional New South Wales, Australia
Source: PLoS Negl Trop Dis. 2024 Aug 5;18(8):e0012385. doi: 10.1371/journal.pntd.0012385 (PMC11326637; doi:10.1371/journal.pntd.0012385)
Supplement: S2 Table — (DOCX) [file pntd.0012385.s002.docx]

S2 Table. NSW postcodes from which samples were tested for Q fever by NSW Health Pathology lab during the same period

|  | | | | | |
| --- | --- | --- | --- | --- | --- |
| Postcode | | Samples received | Percent | Valid Percent | Cumulative Percent |
| Valid | 2251 | 1 | .1 | .1 | .1 |
|  | 2257 | 1 | .1 | .1 | .3 |
|  | 2261 | 2 | .3 | .3 | .5 |
|  | 2265 | 1 | .1 | .1 | .7 |
|  | 2285 | 1 | .1 | .1 | .8 |
|  | 2287 | 1 | .1 | .1 | 1.0 |
|  | 2323 | 1 | .1 | .1 | 1.1 |
|  | 2330 | 1 | .1 | .1 | 1.2 |
|  | 2340 | 35 | 4.8 | 4.8 | 6.0 |
|  | 2350 | 1 | .1 | .1 | 6.2 |
|  | 2358 | 1 | .1 | .1 | 6.3 |
|  | 2360 | 1 | .1 | .1 | 6.4 |
|  | 2365 | 1 | .1 | .1 | 6.6 |
|  | 2381 | 1 | .1 | .1 | 6.7 |
|  | 2388 | 1 | .1 | .1 | 6.8 |
|  | 2425 | 1 | .1 | .1 | 7.0 |
|  | 2439 | 1 | .1 | .1 | 7.1 |
|  | 2440 | 1 | .1 | .1 | 7.3 |
|  | 2443 | 3 | .4 | .4 | 7.7 |
|  | 2444 | 5 | .7 | .7 | 8.3 |
|  | 2446 | 2 | .3 | .3 | 8.6 |
|  | 2447 | 3 | .4 | .4 | 9.0 |
|  | 2449 | 2 | .3 | .3 | 9.3 |
|  | 2450 | 5 | .7 | .7 | 10.0 |
|  | 2452 | 1 | .1 | .1 | 10.1 |
|  | 2456 | 2 | .3 | .3 | 10.4 |
|  | 2460 | 2 | .3 | .3 | 10.7 |
|  | 2463 | 1 | .1 | .1 | 10.8 |
|  | 2470 | 1 | .1 | .1 | 10.9 |
|  | 2480 | 1 | .1 | .1 | 11.1 |
|  | 2500 | 14 | 1.9 | 1.9 | 13.0 |
|  | 2502 | 1 | .1 | .1 | 13.1 |
|  | 2505 | 2 | .3 | .3 | 13.4 |
|  | 2506 | 5 | .7 | .7 | 14.1 |
|  | 2508 | 3 | .4 | .4 | 14.5 |
|  | 2515 | 3 | .4 | .4 | 14.9 |
|  | 2516 | 3 | .4 | .4 | 15.3 |
|  | 2517 | 1 | .1 | .1 | 15.5 |
|  | 2518 | 4 | .5 | .5 | 16.0 |
|  | 2519 | 1 | .1 | .1 | 16.1 |
|  | 2525 | 5 | .7 | .7 | 16.8 |
|  | 2526 | 4 | .5 | .5 | 17.4 |
|  | 2527 | 27 | 3.7 | 3.7 | 21.1 |
|  | 2528 | 21 | 2.9 | 2.9 | 23.9 |
|  | 2529 | 27 | 3.7 | 3.7 | 27.6 |
|  | 2530 | 25 | 3.4 | 3.4 | 31.1 |
|  | 2533 | 25 | 3.4 | 3.4 | 34.5 |
|  | 2534 | 18 | 2.5 | 2.5 | 36.9 |
|  | 2535 | 24 | 3.3 | 3.3 | 40.2 |
|  | 2536 | 6 | .8 | .8 | 41.0 |
|  | 2537 | 3 | .4 | .4 | 41.5 |
|  | 2538 | 9 | 1.2 | 1.2 | 42.7 |
|  | 2539 | 65 | 8.9 | 8.9 | 51.6 |
|  | 2540 | 206 | 28.2 | 28.2 | 79.8 |
|  | 2541 | 92 | 12.6 | 12.6 | 92.3 |
|  | 2548 | 1 | .1 | .1 | 92.5 |
|  | 2550 | 2 | .3 | .3 | 92.7 |
|  | 2575 | 2 | .3 | .3 | 93.0 |
|  | 2576 | 1 | .1 | .1 | 93.2 |
|  | 2577 | 6 | .8 | .8 | 94.0 |
|  | 2582 | 2 | .3 | .3 | 94.3 |
|  | 2594 | 1 | .1 | .1 | 94.4 |
|  | 2602 | 1 | .1 | .1 | 94.5 |
|  | 2611 | 1 | .1 | .1 | 94.7 |
|  | 2643 | 15 | 2.1 | 2.1 | 96.7 |
|  | 2650 | 3 | .4 | .4 | 97.1 |
|  | 2652 | 1 | .1 | .1 | 97.3 |
|  | 2655 | 1 | .1 | .1 | 97.4 |
|  | 2663 | 1 | .1 | .1 | 97.5 |
|  | 2675 | 1 | .1 | .1 | 97.7 |
|  | 2680 | 1 | .1 | .1 | 97.8 |
|  | 2681 | 1 | .1 | .1 | 97.9 |
|  | 2702 | 1 | .1 | .1 | 98.1 |
|  | 2705 | 1 | .1 | .1 | 98.2 |
|  | 2707 | 1 | .1 | .1 | 98.4 |
|  | 2716 | 1 | .1 | .1 | 98.5 |
|  | 2790 | 1 | .1 | .1 | 98.6 |
|  | 2794 | 1 | .1 | .1 | 98.8 |
|  | 2800 | 2 | .3 | .3 | 99.0 |
|  | 2821 | 1 | .1 | .1 | 99.2 |
|  | 2832 | 4 | .5 | .5 | 99.7 |
|  | 2850 | 1 | .1 | .1 | 99.9 |
|  | 2852 | 1 | .1 | .1 | 100.0 |
|  | Total | 731 | 100.0 | 100.0 |  |
